# Acute Exposure to the Cold Pressor Stress Impairs Working Memory Functions: An Electrophysiological Study

**DOI:** 10.3389/fpsyt.2020.544540

**Published:** 2020-11-23

**Authors:** Zengyou Xin, Simeng Gu, Lei Yi, Hong Li, Fushun Wang

**Affiliations:** ^1^Brain and Cognitive Neuroscience Research Center, Liaoning Normal University, Dalian, China; ^2^Institute of Brain and Psychological Sciences, Sichuan Normal University, Chengdu, China; ^3^School of Education Science, Minnan Normal University, Zhangzhou, China; ^4^Department Medical Psychology, Jiangsu University Medical School, Zhenjiang, China; ^5^College of Psychology and Sociology, Shenzhen University, Shenzhen, China

**Keywords:** acute stress, working memory (WM), contralateral delay activity (CDA), prefrontal cortex (PFC), N_2_pc

## Abstract

The results of previous literature focusing on the effects of acute stress on human working memory (WM) are equivocal. The present study explored the effects of acute stress on human WM processing using event-related potential (ERP) techniques. Twenty-four healthy participants were submitted to stressful treatments and control treatment at different times. Cold pressor stress (CPS) was used as stressful treatment, while warm water was used as the control treatment before the WM task. Exposure to CPS was associated with a significant increase in blood pressure and salivary cortisol. After the 3-min resting period, systolic blood pressure (SBP) and diastolic blood pressure (DBP) for the CPS session significantly increased relative to the control treatment session (all *p* ≤ 0.01), and data also showed a significant increase of 20-min post-treatment cortisol concentration (*p* < 0.001) for CPS. Data from the CPS session showed significantly longer reaction times, lower accuracy, and WM capacity scores than that of the control treatment session. Interestingly, a difference between the two sessions was also found in N_2_pc and the late contralateral delay activity (late CDA) components. Specifically, although non-significant main effects of treatment were found for N_2_pc amplitudes, there was a significant interaction between treatments and stimuli conditions (processing load) [*F*_(2,46)_ = 3.872, *p* = 0.028, η2 *p* = 0.14], which showed a pronounced trend toward equalization of N_2_pc amplitude across stimuli conditions during the CPS session clearly different from that of control treatment. As for amplitudes for late CDA, a nearly significant main effect of Treatment was found (*p* = 0.069). That is, the mean amplitude of the late CDA (−2.56 ± 0.27) for CPS treatment was slightly larger than that (−2.27 ± 0.22) for warm water treatment. To summarize, this study not only reported performance impairments in the WM task during CPS trials but also provided high temporal resolution evidence for the detrimental effects of acute stress on processes of information encoding and maintenance.

## Introduction

The acute stress response is associated with a number of neurochemical responses that trigger the release of various hormones and neurotransmitters, which, acting as neuromodulators, can change cellular properties of large-scale neuronal populations throughout the brain (1). We have shown that a stressful event can induce the release of many stress hormones and neuromodulators (2). In recent years, studies have shown that exposure of humans to acute stressors influences cognitive processing and performance [e.g., (3–5)]. Acute stress used in these studies refers to participants' psychobiological response to a brief laboratory stress induction paradigm, such as cold water pressure [also called cold pressor stress (CPS); e.g., (6)], the Trier Social Stress Test [TSST; (7)], short film clips [e.g., (8, 9)], and so on.

Working memory (WM) is responsible for holding a limited amount of information active for a short time span and is also important for optimal functioning of the executive control involved in filtering out irrelevant information, a function that seems to be particularly important under stressful conditions (3, 9). Up until now, some researchers have focused specifically on the effects of acute stress on human WM and the available data are equivocal. Literature shows that human WM performance after acute stress can be impaired (5, 9–14), as well as improved, not affected, or both improved and impaired (3, 6, 8, 15–20).

These studies suggest that acute stress can affect WM, but the impact direction needs to be further clarified. Thus, the main objective of this study was to investigate how acute stress modulates WM process. Several factors may contribute to the inconsistent findings. Firstly, individual differences, such as the gender and the age of the participant, may moderate the findings because of relevant hormonal fluctuations (9, 20, 21). Secondly, the WM task employed may be attributed to the lack of consistency (5, 14, 22). Thirdly, duration, intensity of exposure, and method used to induce acute stress may mediate the effects on WM (5, 14). Other reasons for these discrepancies may include the time course of stress induction or measurement of WM process (3, 9, 13, 14, 22).

According to the attentional control theory [ACT; (23, 24)], WM function is related to the inhibiting efficiency of task-irrelevant information. Improved (or deficient) inhibition control function mainly promotes (or impairs) processing efficiency, which may not be reflected in task performance explicitly. Thus, the focus of this study should further shift to the effects of acute stress on WM process. In many of the studies exploring the stress–WM relationship mentioned above, reaction time (RT) and performance accuracy have been used as the only indices, which have been criticized broadly, as both measure the outcome of processing rather than processing *per se* (24, 25). As far as we know, at least two studies have shown no effects of acute stress on performance, but significant effects on neuroimaging measures with contradictory findings (17, 18), which suggest that acute stress may influence various neural processes and it is imperative to delineate in detail the underlying mechanisms of stress-related WM performance using a variety of methods. However, studies investigating the neural mechanism of acute stress effect on human WM are relatively limited (6, 8, 9, 17, 18, 20). Most of the findings show that acute stress increases human fronto-parietal activity (20) and the regional oxygen saturation of the frontal lobes (6), or results in larger signal change in PFC from baseline (17), or reduces activity in the medial temporal lobe (8) during WM tasks, which are assumed to support WM processing. However, Qin et al. (18) showed that acute stress resulted in significantly reduced WM-related activity in the dorsolateral prefrontal cortex and less deactivation in DMN, which is assumed to disrupt WM processing. Additionally, there are two electrophysiological studies concerning acute stress effect on WM that report inconsistent findings. Gärtner et al. (9) recorded frontal theta activity (4–8 Hz) during the performance of 2- and 3-back tasks, and showed that WM-related frontal theta activity and task performance were decreased under acute stress. Another study concerned only frontal alpha activity and did not report any acute stress effect on parietal alpha power, but a beneficial effect of stress on RT without decreasing accuracy (6). Interestingly, similar to the results of behavioral and neuroimaging studies, the two experiments provided contradictory findings and were carried out by using frequency-domain features of the EEG signal and not specially designed to investigate stress effects on the whole dynamic course of WM processing. Thus, controlling the potential extraneous factors mentioned earlier, by virtue of the high temporal resolution advantages of EEG, analysis of time-domain features of the event-related potentials (ERPs) was used in the present study to directly explore the time course during which acute stress modulates WM processing.

Vogel and colleagues (26–28) described an ERP component, the contralateral delay activity (CDA), with a 300–900-ms window after the onset of the memory array, which was a sustained negative voltage at posterior electrodes during the maintenance phase of WM. The amplitude of the CDA increases with the number of representations held in WM and reaches an asymptote at an individual's WM capacity. Therefore, the CDA can be used as a neurophysiological marker of the numbers of task-relevant and -irrelevant items held in visual WM during the retention interval and can be used to judge how the subject's WM function is going on by comparing the amplitudes for memory arrays with or without distractors.

The paradigm used by Vogel and colleagues was adopted in this study, and ERP components were analyzed that indexed attentional selection of the stimuli [the N_2_pc component; (29)], object identification [the early CDA component; (30)], and the maintenance of WM representations [the late CDA component; (30)].

**Figure 1:**
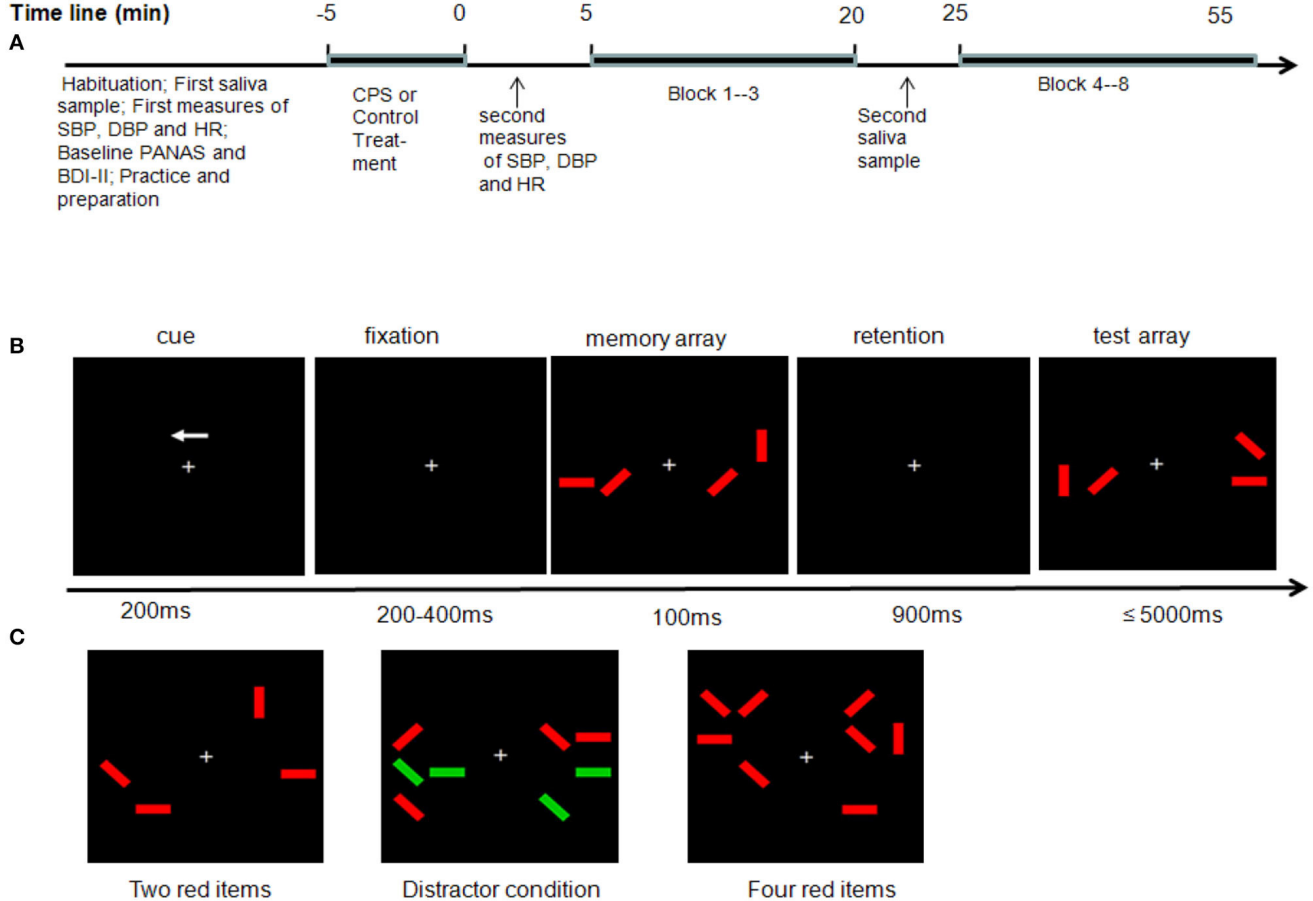
Experimental procedure, stimuli sequence, and array types. **(A)** Timeline of the experimental session. **(B)** Example of two red item conditions in a change trial in which the orientations of the red rectangles in the left hemifield are to be remembered. **(C)** Three task conditions.

Studies have revealed a strong relationship between selective attention and visual WM, such that not only attention is biased by what is on our mind, but selective attention determines what is to be recoded, stored, and processed later (31). Representation of task-relevant information at hand is active and within the focus of central executive processes, which means that it was selected by suppressing the irrelevant and gained full access to be further processed. N_2_pc, a negative component at the posterior sites, is associated with selective attention, reflecting the top-down guided attentional selection toward the relevant information (32) and object individuation (33, 34). Using visual search task, Sänger et al. (35) investigated the effects of acute stress on selective attention and showed that acute stress impaired the attentional allocation and resulted in a reduced N_2_pc. Because participants had no advanced information about the location of the targets under this paradigm, the orienting of attention involved processing competition between goal-driven and stimulus-driven, of which the former was vulnerable to stress exposure. Thus, according to the authors, N_2_pc was decreased significantly for the stressed participants. In the present study, subjects switched their attention to target side in advance (according to cues) and thus N_2_pc component reflected more about processing of object individuation. Mather et al. (36) proposed an “NE hot spots” model, according to which acute stress induces high norepinephrine (NE) level, which can induce fear and anger emotions or fight or flight behaviors (37, 38) and can bias perception and WM in favor of more salient information representations, i.e., stimuli-driven process. In the paradigms of Vogel et al. (28) and Qi et al. (30), distractors are highly salient since their luminance is higher than that of the target items (see Figure 1C). Thus, in the distractor condition, more objects would be involved in the individuation process, and N_2_pc under acute stress would therefore be expected to be greater than under control treatment.

The early and the late CDA components were first proposed by Qi et al. (30), which referred to the early and the late time windows (split at about 450 ms post-stimuli) of the CDA (26–28). Qi et al. (30) proposed that the early CDA might reflect object identification after object individuation, which involved object categorizing in greater detail, and we explored this component as well. The late CDA component in accordance with Vogel et al. (27, 28), was thought to reflect the maintenance of information in WM (30). As reviewed earlier, acute stress improves or impairs WM task performance and the processing of WM-related neural networks. According to the paradigms of Vogel et al. (28) and Qi et al. (30), if it impairs WM processes, an increased amplitude of CDA would be expected because of deficient inhibition control, and as a result, the number of representations would increase due to the coding and storage of task-irrelevant information. However, if the contrary is the case, an intact or slightly smaller amplitude of CDA would be present because of high-efficient inhibition of irrelevant information. According to Luck and Vogel (39), visual WM has a representational limit of about three to four objects. Thus, WM processing in high-load trials of the present study approaches or reaches the limit, while sufficient resources can be allocated under low-load trials (see Figure 1). Therefore, larger CDA amplitude changes would be expected under low-load trials after stress relative to control treatment. Given that behavioral indices reflect the processing outcome rather than processing efficiency, we predict that WM performance would be more prone to be affected by acute stress during the more challenging trials.

## Methods

### Participants

Thirty female volunteers participated in the present study. Two participants were excluded because of excessive eye movements in the cue–target interval, and four other participants dropped out. Thus, data from 24 participants between 18 and 23 years (mean age = 20.1, SD = 1.1 years) were included in the analyses. They were all in good physical health, medication-free, non-smokers, right-handed, and with normal or corrected-to-normal vision. Only women during their luteal phase (with regular menstrual cycles, day 18–25) were included to control possible gender and ovarian cycle effects on adrenocortical reactivity (40, 41). None of them had a history of neurological or psychiatric disorders. Moreover, participants were asked to refrain from caffeine and alcohol within 4 h before the experimental sessions. The volunteers were recruited by announcements and received financial compensation. The study was approved by the Ethics Committee of the Sichuan Normal University. All of them gave their written informed consent prior to their inclusion in the study.

### Procedure and Stimuli

After a participant's arrival, she was allowed to rest briefly, and then a pre-experimental saliva (cortisol) measurement was taken, and systolic blood pressure (SBP), diastolic blood pressure (DBP), and heart rate (HR) were recorded at the same time. Then, participants filled out the Positive and Negative Affect Schedule [PANAS; (42)] and Beck Depression Inventory-Second Edition [BDI-II; (43)], and all participants also filled out the Trait Anxiety Inventory test (44) during their first experimental session. Then, participants were exposed to either the CPS treatment or the warm water control treatment. Immediately after treatment, all subjects had to rest for 3 min and then SBP, DBP, and HR were measured. Then, subjects were engaged in the WM task. Further saliva samples were taken immediately after the third block was finished, about 20 min after the onset of the treatment (Figure 1A).

During the WM task, participants were seated comfortably about 75 cm from a 19-in. screen in an electromagnetically shielded room. They performed a lateralized change detection task, adopted from Qi et al. (30), in which they were cued to remember the visual stimuli on one side of the display and ignore the other side. In each trial (Figures 1B,C), the participants were presented with a brief bilateral array of colored rectangles of varying orientations (vertical, horizontal, left 45°, and right 45°). The stimulus positions and orientations were randomized. The numbers of targets and distractors were the same in both hemifields and only the location of the stimuli could differ between hemifields. The task was to remember the orientations only of the red items and to ignore the green ones in either the left or the right hemifield, depending on the cue presented. Green distractors were more physically salient than the red items, as the luminance of green was higher than that of red. There were three types of stimuli arrays (Figure 1C). In the two-red-items condition, only two red items were shown on each side of the display. In the four-red-items condition, only four red items were shown on each side. In the distractor condition, two red items along with two green distractors were shown on each side.

Each trial began with a 200-ms arrow presented above a fixation cross (Figure 1B). The arrow cued participants to remember the orientations of only red items in either the left or the right side of the memory array. Following a variable interval of 200–400 ms, a memory array was presented for 100 ms. The memory array was removed from the display for 900 ms (retention period). The test array was then displayed for a maximum of 5,000 ms. Participants responded by pressing one of two vertically aligned keys as soon as possible to indicate whether or not a change was present. On one half of the trials, the memory and test arrays were identical, whereas on the other half, the orientation of a single red rectangle within the to-be-remembered side of the memory array was different from its orientation in the test array. Key allocations were counterbalanced between the participants. The instructions emphasized accuracy rather than speed. Moreover, participants were also instructed to keep their eyes fixated throughout the task. The intertrial interval was 2,000 ms. Eight blocks were presented, and each block included 60 trials, in which the three types of memory array were randomly assigned. Overall, participants experienced 160 trials for each type and took ~45 min.

This experiment was conducted by adopting a within-subject design, in which CPS and control treatment were, respectively, applied to subjects by an interval of at least 24 h, and treatment order was counterbalanced. Subjects were instructed to submerge their feet in ice-cold water (4–7°C) for 5 min for the CPS session while in warm water (37–40°C) during control treatment. To avoid any influence of the circadian profiles of adrenocortical reactivity and cognitive ability, CPS and control treatment were conducted in the same time period of the experiment day, and the other experimental procedures were the very same.

The method of salivary cortisol measurement was described in Yang et al. (45). All salivary samples were stored at −40°C, and analyses were completed within about 1 month.

### EEG Recording and Processing

Brain electrical activity was recorded at 64 scalp sites using Ag/AgCl electrodes mounted on an elastic cap (Brain Product, München, Germany), with references on FCz, and a ground electrode on the medial frontal aspect. Vertical electrooculograms (EOGs) were recorded supra- and infra-orbitally at the left eye. The horizontal EOG was recorded from the left vs. right orbital rim. The EEG and EOG were amplified using a 0.05–100-Hz bandpass and were continuously digitized at 1,000 Hz/channel. All interelectrode impedances were maintained below 5 kΩ. Offline, the data were referenced to the average of the left and right mastoids, low-pass-filtered at 30 Hz and a roll-off of 12 dB/octave, segmented (−200 to 900 ms from the onset of the memory array), and baseline-corrected (200 ms). Trials containing saccades (horizontal EOG exceeding ± 30 μV), blinks (Fpz exceeding ± 60 μV, vertical EOG exceeding ± 75 μV), or muscle artifacts (all other electrodes exceeding ± 75 μV) were removed from further analyses. In addition, we performed extra checks according to the procedures described by Qi et al. (30), to assess the potential effects of the residual horizontal-EOG activity on the target ERP components and the data showed that our N_2_pc or CDA results cannot be explained by the residual EOGs. Each correct segment was averaged for each condition separately.

### Measures and Analyses

The primary behavioral measure was WM capacity, an estimation of the amount of retrievable objects. Pashler's formula was used because the task in this study used whole-display probes (30, 46). Specifically, K = N × (HR – FA)/(1 – FA), where K is WM capacity, N is the number of to-be-remembered items, HR is the hit rate, and FA is the false alarm rate. We also computed WM capacity for the distractor condition by filling in 2 for N, because of the two target items (47). In addition, the behavioral measures also included (1) RTs for correct detections and (2) response accuracy.

**Figure 2:**
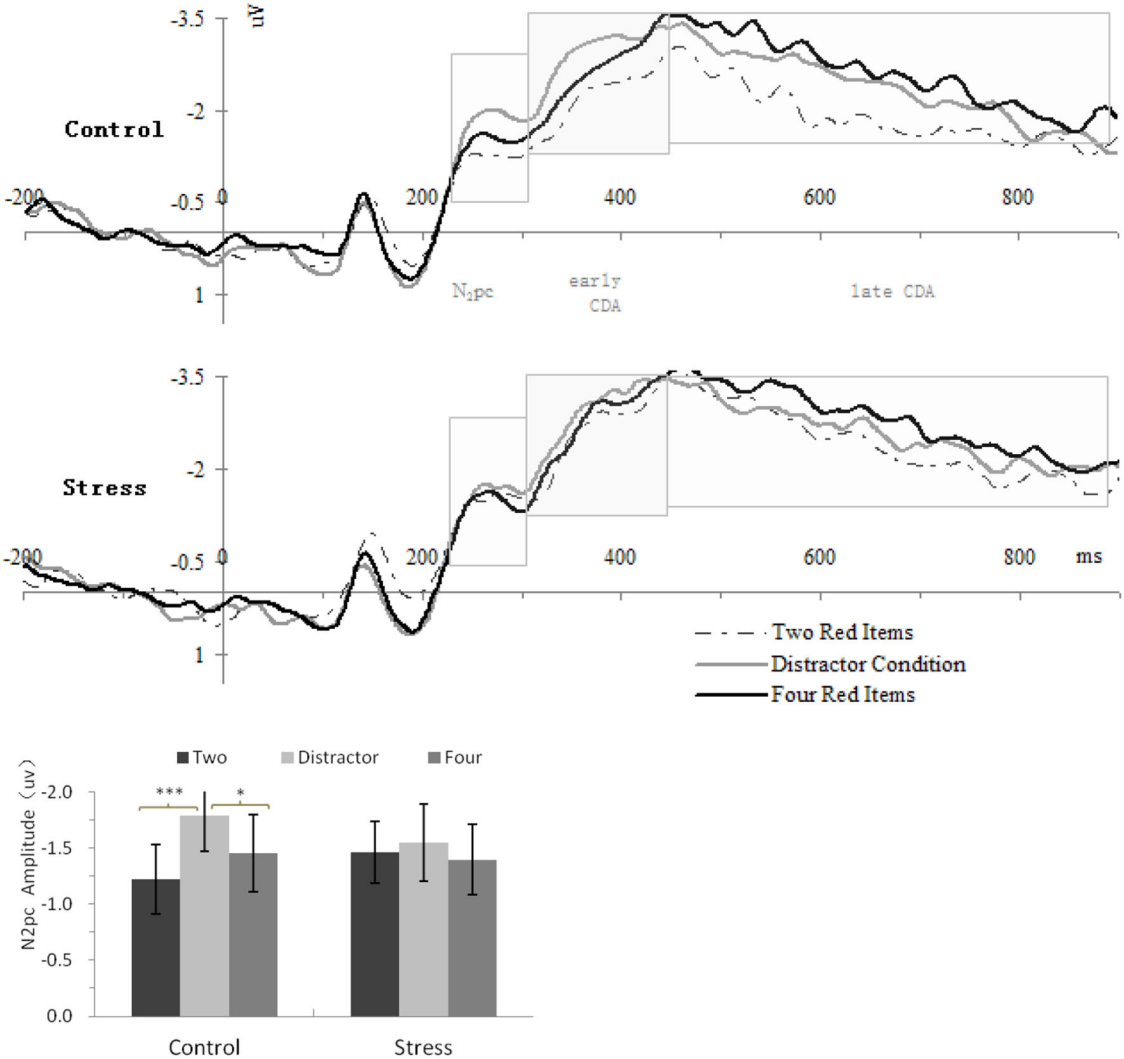
Upper panel, grand-average event-related potential (ERP) waveforms timelocked to memory array onset, showing the contralateral delay activity (CDA) difference waves for each treatment in each condition; Lower panel, mean amplitudes of the N_2_pc component. Error bars represent standard errors of the means. Two, two red items; Distractor, the distractor condition; Four, four red items.*p < 0.05, ***p < 0.001.

The analysis of the underlying neural mechanisms focused on lateralized ERP components elicited by the memory array. The averaged epoch for the ERPs was 1,100 ms, including 200-ms pre-memory-array and 900-ms post-memory-array onset. Separate averages were computed for each participant in each of the three different conditions and for contralaterality (electrode contralateral vs. ipsilateral to the location of memory arrays). Contralateral waveforms were calculated as the average of the left-sided electrodes to the right-sided items and of the right-sided electrodes to the left-sided items. Ipsilateral waveforms were calculated as the average of the left-sided electrodes to the left-sided objects and of the right-sided electrodes to the right-sided objects (28, 30). The lateralized ERP components were then computed by subtracting the mean amplitudes of the ipsilateral waveform from those of the contralateral waveform. On the basis of previous work (28, 30, 48), mean activity from four pairs of lateral posterior electrode sites (P3/4, P5/6, PO3/4, and PO7/8) were used to calculate the lateralized ERP components. Figure 2 depicts the lateralized ERP waveforms for the three conditions and the two treatments separately at these electrode pairs. Given the previous studies (28, 30, 48) and our data, three measurement windows were selected: 230–310 ms (N_2_pc component), 310–450 ms (early-CDA component), and 450–900 ms (late CDA component) after the onset of the memory array, and the resulting mean amplitudes were calculated for further analysis.

In order to meet the normality assumption according to Shapiro–Wilk test, we conducted logarithmic transformation for data of emotion questionnaires and Box–Cox transformation for all electrophysiological data. Then, all the data were assessed using repeated-measures ANOVA by SPSS version 19.0, and necessary adjustments were made where assumptions were violated (e.g., by using Greenhouse–Geisser degrees of freedom corrections).

## Results

### Mood, Trait Anxiety, and Physiological Measurements

To evaluate potential differences in mood variables between the CPS and the control sessions, and effects of stress induction, repeated-measures ANOVA was conducted with Treatment (CPS vs. control) as a repeated measure (Table 1). The ANOVA showed no difference for mood, basal HR, basal blood pressure, and basal cortisol concentration between the CPS and control sessions. However, results showed significant effect of treatment for post-resting blood pressure. That is, after the 3-min resting period, SBP and DBP for the CPS session significantly increased relative to the control treatment session (all *p* = 0.01). Data also showed significant effect of treatment for 20-min post-treatment cortisol concentration (*p* < 0.001). Moreover, results of anxiety test demonstrated that all participants are of moderate level of trait anxiety (mean = 42.2, SD = 6.2).

**Table 1:** Pre-experiment mood and physiological measurements before and after control or CPS treatment.

	**Positive affect**	**Negative affect**	**Depression**	**Baseline HR**	**HR after resting 3 min**
CON (*M, SD*)	26.2 (9.7)	14.5 (6.4)	6.8 (5.0)	79.6 (8.2)	75.6 (7.5)
CPS (*M, SD*)	27.1 (8.5)	16.0 (6.5)	7.2 (5.3)	80.7 (11.7)	75.2 (9.6)
ANOVA (*F, p*)	0.8 (0.38)	2.6 (0.12)	0.2 (0.67)	0.2 (0.64)	0.1 (0.81)
**Baseline SBP**	**SBP after resting 3 min**	**Baseline DBP**	**DBP after resting 3 min**	**Baseline Cortisol (nmol/L)**	**Cortisol after 20 min (nmol/L)**
101.2 (6.4)	98.7 (9.0)	63.5 (7.5)	62.3 (6.1)	3.1 (1.1)	3.1 (1.2)
102.8 (5.4)	105.2 (6.3)	64.0 (6.0)	66.0 (7.0)	3.1 (1.1)	6.7 (2.0)
2.5 (0.13)	7.0(0.01)	0.1 (0.73)	7.4 (0.01)	0.1 (0.75)	75.1 (0.00)

*Values represent means (M) and standard deviations (SD); CON, control treatment; CPS, cold pressor stress; HR, heart rate (beats per minute); DBP, diastolic blood pressure (mmHg); SBP, systolic blood pressure (mmHg); CORT, cortisol (nmol/L)*.

### Behavioral Results

Repeated-measures ANOVAs were conducted with two within-subject factors: Treatment (CPS vs. control) and Condition (two red items, four red items, and the distractor condition), focusing on WM capacity scores, RT for correct detections, and accuracy. The ANOVA showed significant main effects of Treatment [*F*_(1,23)_ = 9.39, *p* = 0.005, η2 *p* = 0.29] and Condition [*F*_(2,46)_ = 141.97, *p* = 0.000, η2 *p* = 0.86], and interaction of Treatment × Condition [*F*_(2,46)_ =3.65, *p* = 0.034, η2 *p* = 0.14] for WM capacity. Further analysis showed significant differences between CPS and control treatment only in four red items (2.53 ± 0.09 vs. 2.70 ± 0.09, *p* = 0.029) and distractor condition (1.53 ± 0.03 vs. 1.61 ± 0.02; *p* = 0.002). WM capacity for two red items also decreased after CPS treatment but did not reach statistical significance (*p* = 0.818). Repeated-measures ANOVA showed significant main effects of Treatment [*F*_(1,23)_ = 11.34, *p* = 0.003, η2 *p* = 0.33] and Condition [*F*_(2,46)_ = 252.17, *p* = 0.000, η2 *p* = 0.92], and interaction of Treatment × Condition [*F*_(2,46)_ = 3.78, *p* = 0.03, η2 *p* = 0.14] for accuracy. Further analysis indicated significant differences between CPS and control treatment only in four red items (0.79 ± 0.01 vs. 0.81 ± 0.01, *p* = 0.035) and distractor condition (0.86 ± 0.007 vs. 0.90 ± 0.007; *p* = 0.001). There was no statistical significance (*p* = 0.464) for two red items. As for RTs, the ANOVA showed significant main effects of Treatment [*F*_(1,23)_ = 5.28, *p* = 0.031, η2 *p* = 0.19] and Condition [*F*_(2,46)_ = 66.87, *p* = 0.000, η2 *p* = 0.74]. Further analysis indicated that RTs (814 ms) for CPS was significantly longer than that (747 ms) for control treatment. The RTs was shortest for the two red items (742 ms), followed by distractor condition (777 ms), and then four red items (821 ms; for all *p* < 0.001). Further analysis also showed significant differences between CPS and control treatment in all conditions: *p* = 0.030 for four red items (856 vs. 785 ms), *p* = 0.049 for distractor condition (809 vs. 746 ms), and *p* = 0.023 for two red items (776 vs. 708 ms).

### Lateralized ERP Results

Repeated-measures ANOVAs were conducted with two within-subject factors: Treatment (CPS vs. control) and Condition (two red items, four red items, and the distractor condition), to evaluate potential differences in ERP amplitudes between treatments and among different stimuli conditions.

N_2_pc (230–310 ms)

Figure 2 (lower panel) shows the N_2_pc amplitude as a function of Condition and Treatment. Repeated-measures ANOVA showed a significant main effect of Condition [*F*_(2,46)_ = 3.725, *p* = 0.032, η2 *p* = 0.14] and interaction of Treatment × Condition [*F*_(2,46)_ = 4.507, *p* = 0.016, η2 *p* = 0.164] for N_2_pc. Concerning the interaction of Treatment × Condition, repeated-measures ANOVAs conducted, respectively, within each treatment indicated that the N_2_pc amplitudes for control treatment differed significantly across conditions, *F*_(2,46)_ = 8.421, *p* = 0.001, η2 *p* = 0.27, but there were no significant difference across conditions for CPS, *F*_(2,46)_ = 0.38, *p* = 0.61, η2 *p* = 0.02. *Post-hoc* analyses for control treatment showed that the N_2_pc amplitude for the distractor condition (−1.79 ± 0.316) was larger than that for four red items (−1.45 ± 0.344) (*p* = 0.017) and for two red items (−1.22 ± 0.309) (*p* = 0.001).

Early CDA (310 to 450 ms) (Table 2).

**Table 2:** Early and late CDA amplitudes for cold pressor stress (CPS) treatment and control treatment across three conditions (M ± SD).

	**Two red items**		**Distractor condition**		**Four red items**	
	**Stress**	**Control**	**Stress**	**Control**	**Stress**	**Control**
Early CDA	−2.65 (1.48)	−2.24 (1.56)	−2.95 (2.04)	−2.96 (1.67)	−2.78 (1.72)	−2.65 (1.77)
Late CDA	−2.38 (1.26)	−1.89 (1.00)	−2.54 (1.62)	−2.34 (1.18)	−2.77 (1.42)	−2.58 (1.30)

**Figure 3:**
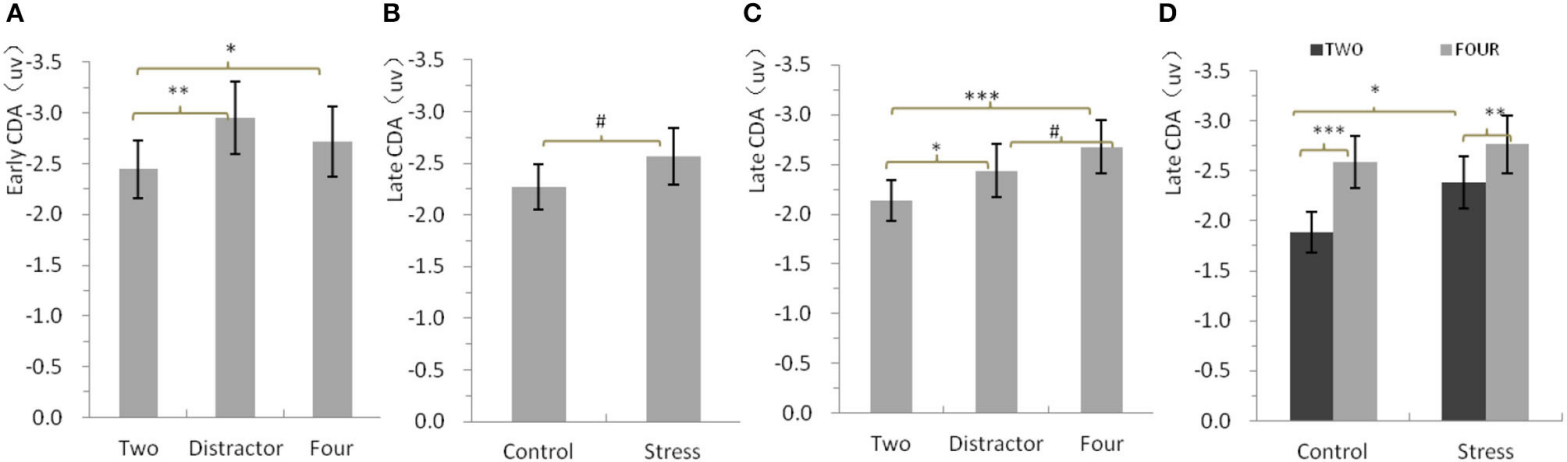
**(A)** Mean amplitudes of the early contralateral delay activity (CDA) potential, between 310 and 450 ms after memory array onset. **(B–D)** Mean amplitude of the late CDA, between 450 and 900 ms after memory array onset. Error bars represent standard errors of the means.*p < 0.05, **p < 0.01, ***p < 0.001, #*p* = 0.069 for b, #*p* = 0.052 for c.

Figure 3A shows the mean amplitudes of the early CDA as a function of conditions. Repeated-measures ANOVA only showed a significant main effect of Condition [*F*_(2,46)_ = 5.97, *p* = 0.009, η2 *p* = 0.21] for the mean amplitudes of the early CDA. Concerning the main effect of condition, larger early CDA amplitudes were found for the distractor condition and four red items with respect to two red items (*p* = 0.003 and 0.019, respectively), while there was no significant difference between the distractor condition and the four red items (*p* = 0.186).

Late CDA (450 to 900 ms) (Table 2).

Figures 3B–D show the mean amplitudes of the late CDA as a function of all conditions. Repeated-measures ANOVA showed a significant main effect of Condition [*F*_(2,46)_ =11.44, *p* = 0.000, η2 *p* = 0.332] and a nearly significant main effect of Treatment [*F*_(1,23)_ = 3.63, *p* = 0.069, η2 *p* = 0.136] for the mean amplitudes of the late CDA. Further analysis indicated that the mean amplitudes of the late CDA of all three types of stimuli arrays (−2.56 ± 0.27) for CPS treatment were nearly significantly larger than that (−2.27 ± 0.22) for control treatment (Figure 3B). Concerning the main effect of condition, larger late CDA amplitudes were found for the four red items and the distractor condition with respect to two red items (*p* = 0.000 and 0.027, respectively), while larger late CDA amplitudes were also found for four red items with respect to the distractor, but the difference was borderline significant (*p* = 0.052; Figure 3C). Given the concerns of this study and the attempts to make clear how stress affects the maintenance in visual–spatial WM, we conducted repeated-measures ANOVAs with two red items and four red items as one factor and Treatment as another one. The results showed that CPS significantly increased the amplitudes of late CDA [*F*_(1,23)_ = 4.38, *p* = 0.047, η2 *p* = 0.16], especially for the two items (*p* = 0.026), and the same as mentioned above, the amplitudes for four red items were much larger than those for two red items [*F*_(1,23)_ = 43.07, *p* = 0.00, η2 *p* = 0.65], with no interaction (Figure 3D).

## Discussion

The main objective of this study was to investigate the effect of acute stress on WM processing. In the present study, we used a well-validated neural measure of visual–spatial WM storage to investigate how acute stress influences human WM functions. The main findings were that the subjects after control treatment performed the visual WM task better than after the CPS treatment, and as expected, subjects showed a late CDA amplitude in the two-red-items condition after the CPS treatment, which was significantly larger than that after the control treatment, but no significant difference in the four-red-items condition and distractor condition between the treatments. More importantly, as for the ERP waves, it was statistically proven that the N_2_pc components showed significant disparity across three types of stimuli arrays after control treatment, which disappeared in the CPS session. These results demonstrate that acute stress impaired individuals' cognition processes of object individuation (as reflected in alterations in N_2_pc amplitude) and maintenance in visual–spatial WM (as reflected in alterations in Late CDA amplitude), as well as in task performance. Therefore, the findings of the present study extend the recent literature investigating the effects of acute stress on human WM processing by providing evidences of detrimental effects of acute stress on WM task performance and the temporal course of the detrimental effects at least as early as the N_2_pc time window using the ERP technique.

To characterize the response to the CPS, salivary cortisol, blood pressure, and HR were assessed. The results revealed significantly higher ratings of blood pressures in the CPS treatment session, as well as an increased activity of the HPA, compared to the control treatment session. These findings are well in line with previous studies [e.g., (6, 14, 17, 49)] and indicate the successful induction of a neuroendocrine stress response.

The findings of longer RTs and accuracy decrement clearly observed in high cognitive load trials under acute stress treatment are in line with our hypothesis and some previous studies [e.g., (5, 9, 11, 14)]. The behavioral data also showed larger WM capacity in high cognitive load trials during the control treatment session with respect to the CPS session. Importantly, our experimental design controlled for a number of extraneous variables, such as individual differences, circadian profiles of adrenocortical reactivity and cognitive ability, and so on, by use of within-subject design and only women during their luteal phase participating in the experiment, and the CPS or control treatment assigned at the same timing of different experimental days, which proved that acute stress impaired individuals' task performance.

Attentional selection was quantified as the magnitude of the N_2_pc component, a well-established ERP component that reflects the focusing of attention onto multistimulus arrays (30, 33, 34). The data of the control session in this study showed clear N_2_pc components for the three conditions, and the mean amplitude of the N_2_pc for the distractor condition was the largest, followed by four red items, and then two red items, which was in accordance with a recent study using the same experimental protocol (30) and supported the proposition that the N_2_pc increased as a function of target numerosity, reflecting a process of object individuation (30, 33, 34). However, the data of the CPS session in this study showed a very different N_2_pc response pattern, which is a pronounced trend of increased amplitude for the two-item condition relative to control treatment, while amplitude was decreased in the distractor condition. Given that Qi et al. (30) using University students as subjects in normal conditions, which was the same as the control treatment condition in this study, reported the same N_2_pc response pattern, CPS treatment substantially impaired subjects' attentional selection and the object individuation process.

However, contrary to our hypothesis, no significantly larger N_2_pc amplitude was shown after CPS. According to Mather et al. (36), acute stress should affect attentive selection, which involved the mechanisms of amplified activation of the irrelevant information representations. Therefore, though no competition between goal-driven and stimulus-driven processes existed, much more task-irrelevant information would be expected to be encoded, leading to a larger N_2_pc component. Some evidence suggests that the N_2_pc can be decomposed into two functionally distinct lateralized subcomponents: the target negativity (Nt), reflecting target selection and object individuation, and the distractor positivity (Pd), associated with distractor suppression [e.g., (50)]. Thus, the outcome pattern under CPS in the present study might result from the increasing input of task-irrelevant information and the disproportionate allocation of resources in the suppression of task-irrelevant information. According to the “NE hot spots” model (36), acute stress triggers locus coeruleus and induces increased NE (2, 51), which biases perception and memory in favor of more salient information representations at the expense of less salient representations. At the same time, acute stress also affects PFC network by the induction of catecholamines and cortisol (1, 52–55), which is an important regulator of locus coeruleus output and an important area of appraising sensory information based on goal relevance (36). Thus, acute stress might lead to inputting information more based on bottom-up salience, instead of top-down goals. In the case of the present study, acute stress resulted in representations of more task-irrelevant information (i.e., Nt increases significantly), and for the two-red-items condition, there was no need to recruit much resources to suppress irrelevant information (i.e., Pd, would not show a significant change) to perform successfully due to low processing load. Thus, the overlaying of the two subcomponents resulted in N_2_pc amplitude increasing. For the same reasons, during the CPS session, much more resource must be recruited to suppress the task-irrelevant information (i.e., Pd increases significantly) in order to achieve good performance in four-red-item trials and the distractor condition trials in which too much resources are devoted to the object individuation process, and thus, the overlaying of the two subcomponents resulted in N_2_pc amplitude decreasing or no significant change, and in the end, there is no difference in N_2_pc amplitudes across the three types of stimuli arrays in CPS trials. Studies have shown that not only attention is biased by representations in WM, but selective attention determines what is to be stored and processed later (31). Thus, CPS might make more additional task-irrelevant information be encoded during the N_2_pc time window and further processed in WM.

As for the early CDA component, Qi et al. (30) proposed it as a measure of the amount of processing resources required to perform object identification after object individuation, in order to categorize objects in greater detail. Our results showed only the main effect of condition in the early CDA during the time interval of 310–450 ms. Specifically, subjects showed more consumption of processing resources for object identification for the distractor condition and the four red items than that for the two red items, mostly because of identification or categorizing process involving more details in the distractor and the four-red-items conditions, as reflected by their larger early CDA for the distractor condition and the four red items than for the two red items, which is in line with the findings of Qi et al. (30). Our data also showed no main effect of treatment and no interaction of Treatment × Condition, and one possible explanation for this finding is that, in contrast to the control treatment session, more task-irrelevant information was encoded and represented but the classification and identification process might be relatively more difficult during the CPS session. Given the limitations of processing capacity during stimulus classification and identification, and finer processing involved in the control treatment session, no main effect of treatment and interaction was found in the end. If this were true, information maintained in WM during the CPS session were overload and low accuracy. Further studies would be necessary to determine the cognitive correlates and the stress effect on this component, and examination of the early CDA was exploratory in this study.

During the subsequent WM maintenance phase, as expected, the results showed that larger late CDA amplitudes were found for the four red items and the distractor condition with respect to two red items in the control treatment session, which is similar to former studies (28, 30, 47). Analyses of data from CPS and control treatments showed that there was a borderline significant main effect of treatment, in which although significantly larger late CDA amplitudes were found only for two red items during the CPS session with respect to the control treatment session, there was a clear trend of increased late CDA amplitude across all other conditions in the CPS session compared to the control treatment session. These findings supported our hypothesis that larger CDA amplitude changes would appear mostly under low-load trials. Given that there were significant main effects of Treatment in behavioral data and the discussions about N_2_pc and early CDA mentioned earlier, these late CDA findings indicated that acute stress impaired the encoding of information into WM and disrupted the inhibition of irrelevant information in WM via intricate mechanisms, which probably involved the interaction of NE and cortisol and the dysfunction of top-down appraisal of inputting information [see (36)] and PFC network [see (1, 53, 54)]. Thus, more task-irrelevant information got stored in WM due to low-efficient inhibition of irrelevant information, and late CDA amplitudes increased markedly for two red items during the CPS session relative to that of the control session, while only showing an increasing trend for four-red-items and the distractors condition mostly because of the limitation of WM capacity, which was thought to be approximately three to four objects (39).

The finding of detrimental effects of acute stress on WM was in line with some aforementioned studies (5, 9–14). Gärtner et al. (9) investigated the frequency-domain features of the EEG while subjects performed WM tasks, and the results showed impairment effects of acute stress only on the late time window, that is, WM maintenance processing. Although they distinguished two separate time windows (0–800 ms and 1,000–1,800 ms) to calculate the frontal theta power, which was thought to be related to attentional and maintenance processes during WM, respectively, the research design was far from direct observation of the dynamic course of the WM process. However, in this study, we used time-domain features of the ERPs, which could provide the direct observation of the dynamic course of WM processing, by which we discerned that the effects of acute stress emerged about 200 ms after the onset of memory array, and determined that the acute stress effect appeared as early as the end of the N_2_pc time window, which would be difficult to distinguish in studies using frequency-domain feature analysis (6, 9). As far as we know, this study was among the first ones that used time-domain analysis of the ERPs to investigate the effects of acute stress on human WM process and not only reported performance impairments but also provided high temporal resolution evidence for the detrimental effects of acute stress on WM processes.

However other studies reported inconsistent results in the WM task performance, some of which found no or improved effects of stress treatment on WM [e.g., (16–19)]. As mentioned earlier, major explanations for this contradiction might include the following:

1. The different load placed on WM. Three of the aforementioned studies reported acute stress impaired WM at high loads or involving more cognitive operations, but not at low loads (9, 11, 14), and studies reporting no detrimental effects were usually low load, such as the 0-back and 2-back task (8, 18). Sufficient resources can be reallocated in low-load tasks, when additional irrelevant information was represented in WM for exposure to stressor, while in case of high-load tasks, process resources were easily exhausted, as was proved in our study that significant acute stress effects for WM capacity and accuracy for four red items and the distractor conditions were reported, but not for two red items, and the electrophysiological evidence that reflected the employment of resources seemed to be the opposite, that significantly larger late CDA amplitudes were found only for two red items during the CPS session relative to the control session due to limitations of resources.

2. The time interval between assessing WM process and stressor offset. In the aforementioned studies showing harmless stress effects on WM, the cortisol peak was already exceeded when subjects performed the WM task 20 or 30 min after stress procedure [e.g., (3, 6, 15, 16)] and the catecholamine (NE and dopamine) concentration, which is the marker of the activation of the sympathetic nervous system (SNS), had by then returned to baseline, while in studies reporting harmful effects, the WM was tested in < 10 min after the stress exposure [5 min in our experiment; (5, 9–14)] when cortisol levels were rising and SNS might still be activated. Given evidence from animal studies showing that corticosteroid actions interact with catecholaminergic activity and potentiate the negative effects of stress hormones on PFC (56, 57), the detrimental effects of acute stress might be also stronger during concurrent SNS and HPA activation for humans.

3. The intensity or the methods of inducing acute stress. In studies reporting beneficial effects on performance or WM-related networks, subjects were exposed to CPS only for 1 or 2 min (6, 17), or other milder stressors, such as negative movie clips (8), so that the levels of stress hormones secreting might be relatively low. Pharmacological studies in animals have revealed that catecholamines exert an inverted U influence on PFC in which sub- or supra-optimal levels of catecholamine impaired the prefrontal network processing (58, 59), and on the cellular level, a study using intracellular recordings indicated that catecholamine also showed such inverted U relationships with neural firing activities of the dlPFC (60). Similar results were also reported for corticosterone in animals (61). Therefore, the relatively low levels of cortisol or catecholamines might have just reached an optimal point or returned to baseline (in case of the second explanation), resulting in enhanced or normal WM-related neural network activities and performance.

However, as there were no manipulations of stress severity and time interval between WM test and stressor offset in this study, additional studies are required to test the last two hypotheses.

## Limitations

Several limitations of the present study should be noted. In the current experiment, we only use self-reported information to choose participants in the luteal phase, which is characterized by high estradiol and progesterone concentrations. These self-reported measures are problematic, since they might not match rigorous neuroendocrine measurements (21). Future studies should use physiological measures to choose subjects of specific menstrual cycle phase to replicate the present findings and investigate possible differences between different cycle phases, since, for the long-term memory, evidence has been reported that beneficial effects on memory consolidation only occur in the luteal phase (62). Moreover, our study did not consider the influence of other factors (personality traits, intelligence, socioeconomic status, etc.) that may influence WM function, and it will be important to control these factors in future studies.

## Conclusions

To summarize, this study indicates that acute stress has substantial and detrimental effects on WM processing and can lead to large amounts of task-irrelevant information encoded and stored in WM, and the detrimental effects emerge at least as early as the N_2_pc time window.

## Data Availability Statement

The raw data supporting the conclusions of this article will be made available by the authors, without undue reservation.

## Ethics Statement

The studies involving human participants were reviewed and approved by Ethics Committee of the Sichuan Normal University. The patients/participants provided their written informed consent to participate in this study.

## Author Contributions

ZX, LY, and HL planned the study. ZX, SG, and FW analyzed the data. ZX and HL did the writing. All authors contributed to the article and approved the submitted version.

## Conflict of Interest

The authors declare that the research was conducted in the absence of any commercial or financial relationships that could be construed as a potential conflict of interest.
